# A pilot and feasibility randomised controlled study of Prolonged Exposure Treatment and supportive counselling for post-traumatic stress disorder in adolescents: a third world, task-shifting, community-based sample

**DOI:** 10.1186/s13063-016-1677-6

**Published:** 2016-11-17

**Authors:** Jaco Rossouw, Elna Yadin, Debra Alexander, Irene Mbanga, Tracy Jacobs, Soraya Seedat

**Affiliations:** 1 Stellenbosch University, Stellenbosch, Western Cape South Africa; 2 Department of Psychiatry, University of Pennsylvania, Philadelphia, PA USA; 3 Centre for Cognitive-Behaviour Therapy, 67 Visagie Street, Monte Vista, 7460 Western Cape South Africa

**Keywords:** Treatment outcome, Post-traumatic stress disorder, Prolonged exposure, Supportive counselling, Randomised controlled trial

## Abstract

**Background:**

There is a dearth of empirical evidence on the effectiveness of pharmacological and nonpharmacological treatments for adolescents with post-traumatic stress disorder (PTSD) in developing country settings. The primary aim of this study was to demonstrate that Prolonged Exposure Treatment for Adolescents (PE-A) and supportive counselling (SC) are implementable by nurses in a South African context. A secondary aim was to perform a preliminary analysis of the effectiveness of registered nurses delivering either PE-A or SC treatment to adolescents with PTSD. It is hypothesised that PE-A will be superior to SC in terms of improvements in PTSD symptoms and depression.

**Method:**

A pilot, single-blind, randomised clinical trial of 11 adolescents with PTSD. Nurses previously naïve to Prolonged Exposure (PE) Treatment and SC provided these treatments at the adolescents’ high schools. Data collection lasted from March 2013 to October 2014. Participants received twelve 60–90-min sessions of PE (*n* = 6) or SC (*n* = 5). All outcomes were assessed before treatment, at mid-treatment, immediately after treatment completion and at 12-month follow-up. The primary outcome, PTSD symptom severity, was assessed with the Child PTSD Symptom Scale–Interview (CPSS-I) (range, 0–51; higher scores indicate greater severity). The secondary outcome, depression severity, was assessed with the Beck Depression Inventory (BDI) (range, 0–41; higher scores indicate greater severity).

**Results:**

Data were analysed as intention to treat. During treatment, participants in both the PE-A and SC treatment arms experienced significant improvement on the CPSS-I as well as on the BDI. There was a significant difference between the PE-A and SC groups in maintaining PTSD and depression at the 12-month post-treatment assessment, with the participants in the PE-A group maintaining their gains both on PTSD and depression measures.

**Conclusion:**

The treatment was adequately implemented by the nurses and well-tolerated by the participants. Preliminary results suggest that the delivery of either intervention led to a significant improvement in PTSD and depression symptoms immediately post treatment. The important difference was that improvement gains in PTSD and depression in the PE-A group were maintained at 12-month follow-up. The results of this pilot and feasibility study are discussed.

**Trial registration:**

Pan African Clinical Trials Registry: PACTR201511001345372, registered on 11 November 2015.

## Background

Post-traumatic stress disorder (PTSD) is a mental health condition, characterised by intrusive re-experiencing, pervasive avoidance, and hyperarousal symptoms, which some individuals develop as a result of experiencing or witnessing a life-threatening traumatic event. If left untreated, PTSD often develops into a chronically debilitating condition with substantial functional impairment. The scientific literature on exposure to trauma suggests that adolescents seem to be at greatest risk and that PTSD diagnosis peaks in late adolescence [1]. According to this review that examined 32 studies conducted around the world and published between 2000 and 2011 [1], the prevalence rate of PTSD among adolescents ranges widely between 3% (due to natural disaster) to as high as 57% (due to sexual assault), with an average rate of 13.6% and an average age of 15 years. The types of trauma that adolescents are exposed to vary, but commonly include witnessing violence, or experiencing physical or sexual assault.

South Africa is a country with high rates of trauma exposure. In a study conducted in South Africa and Kenya, 14.5% of adolescent students met the criteria for PTSD and an additional 10.3% met partial criteria for PTSD within South Africa [2]. A more recent study examining the rates of PTSD in grade-8 adolescents (14–15 years old) within the greater Cape Town area showed a prevalence of 21% [3].

Depressive conditions, ranging from demoralisation to major depression, are among the most common secondary symptoms of PTSD [4–6]. It has been reported [7] that 41% of adolescents with PTSD meet the criteria for major depression by age 18, compared to 8% among their peers, and that PTSD was associated with a significantly increased risk for social anxiety (33%), specific phobia (29%), alcohol dependence (46%), and drug dependence (25%). Within the Cape Town-based study mentioned above [3], the prevalence of depression was 41% and anxiety was 16%.

Most low- and middle-income countries suffer serious resource shortages [8]. This is also true for South Africa, where the ratio of 0.32 psychologists and 0.28 psychiatrists for every 100,000 people [9] presents a substantial treatment-seeker to treatment-provider gap. Those unable to access private care suffer further since there is a 75% probability that they will not receive the professional attention that they require [10]. It is, therefore, crucial to address the discrepancy between need for, and availability of, psychological interventions by making them more readily available to a broader population.

The World Health Organisation (WHO) has proposed that mental health care services and interventions be shifted to the primary care level [11, 12]. Task-shifting psychosocial interventions from specialised to nonspecialised health workers for treatment of mental disorders has also been proposed as a strategy for expanding access to mental health care where it is needed most [13]. With this in mind, nurses were identified as suitable primary health care workers for the study since they are already working within a community setting.

Given the considerations described earlier, successful dissemination of treatments for PTSD in developing countries requires that a treatment (1) have solid evidence for efficacy for a wide range of traumas, (2) be effective for patients with varied demographic backgrounds, (3) be effective for patients with complex presentations and comorbid diagnoses, (4) be relatively simple and streamlined to facilitate training to a broad base of practitioners with varied qualifications, and (5) have a structured manual that contains a step-by-step guide for the therapist [14].

Prolonged Exposure (PE) Treatment is a trauma-focussed, exposure-based treatment for adults with PTSD following diverse types of traumas with great empirical support, including in a large number of randomised controlled trials (RCTs) [15]. PE is a theory-driven treatment that is based on Emotional Processing Theory [16] which conceptualises chronic PTSD as a failure to process the traumatic memory due to a strong tendency to avoid trauma reminders and the consequential erroneous beliefs about the world being extremely dangerous and about the self as being extremely incompetent. To facilitate recovery from PTSD, PE treatment encourages healthy confrontation with the traumatic memory, both by gradual exposure to real-life triggers and by prolonged and repeated exposure in imagination to the story of the traumatic memory. Prolonged and repeated exposures allow processing of the themes that arise during the retelling, and help patients with PTSD to examine more closely what happened, how they have been affected and why they can recover. This approach facilitates the realisation that thinking about a previously dangerous situation or being reminded of it through triggers is not the same as actually being in the situation itself, and results in sufferers having access to the corrective experiences that enable them to adjust their cognitions to be more realistic, which in turn, improves functioning.

Due to the relative ease of training, PE has been widely disseminated around the world and, following a relatively brief training, nonspecialists have been taught to successfully provide PE [15, 17]. PE for adults has been shown to be effective within a community setting [18] and meets the requirements for dissemination. Prolonged Exposure Treatment for Adolescents (PE-A), which was based on the adult protocol, also appears to meet these requirements. PE-A has already been shown to be effective in community settings in the USA [19] and in Israel [20]. These two studies compared PE-A with another active treatment. The comparative treatment in the USA study was supportive counselling (SC). That study task-shifted treatment to experienced rape counsellors with a Masters’ degree working in a community clinic. The counsellors had been trained in SC but were initially naive to PE-A. The results from that study indicated that the counsellors were effective at implementing PE-A. The participants’ PTSD and depression symptoms improved significantly and were maintained at a 12-month follow-up. The study conducted in Israel compared PE-A with a psychodynamic structured treatment. This study also found PE-A to be effective in the treatment of PTSD. No task-shifting was included in the study.

A pilot and feasibility RCT was initiated with registered nurses trained to provide adolescents suffering from PTSD with either PE-A or SC. It was hoped that the feasibility pilot study would demonstrate that the treatment models would be implementable in a South African context and when provided by nurses. It also gave us the opportunity to identify any possible difficulties with the assessment tools and to obtain preliminary results on the effectiveness of the treatments implemented. We hypothesised that PE-A would be superior to SC in reducing interviewer-assessed PTSD severity and self-reported depression when measured immediately after completion of treatment and at a 12-month follow-up. The present paper reports the outcome of the pilot study only.

## Methods

### Assessment measures

The Child Post-traumatic stress disorder Symptom Scale–Interview (CPSS-I) is a clinician interview that assesses PTSD diagnosis and symptom severity for ages 8 to 18 years. Scores range from 0 to 51 (0–10, below threshold; 11–15, subclinical; 16–20, mild; 21–25, moderate; 26–30, moderately severe; 31–40, severe; 41–51, extremely severe). It has excellent internal consistency (Cronbach *α* = .83–.89) and test-retest reliability (.84–.86). Convergent validity and discriminant validity are high [21, 22].

The Child PTSD Symptom Scale–Self-Report (CPSS-SR) is a scale completed by the patient and has psychometric properties that are equivalent to those of the CPSS-I. Diagnostic agreement between the CPSS-SR and CPSS-I is excellent (85.5%) [21, 22].

The Beck Depression Inventory (BDI) is a self-report that evaluates depression severity [23]. Scores range from 0 to 41 (0–9 not depressed, 10–18 mild, 19–29 moderate, 30–63 severe). It has good internal consistency (Cronbach *α* = .73–.92), split half-reliability is .93, and correlations with clinician ratings of depression range from .62 to .65 [23].

The Mini International Neuropsychiatric Interview for Children and Adolescents (MINI-KID), is a short, structured diagnostic interview for the *Diagnostic and Statistical Manual of Mental Disorders, 4th edition* (DSM-IV) and the *International Classification of Diseases, version 10* (ICD-10) psychiatric disorders in children and adolescents [24]. It has excellent test-retest reliability (0.64–1.00). Substantial to excellent MINI-KID to Schedule for Affective Disorders and Schizophrenia for School-Aged Children–Present and Lifetime Version (K-SADS-PL) concordance was found for the diagnosis of any mood disorder, anxiety disorder, substance abuse disorder, and any behavioral disorder (0.81–0.96) [24]. It was used as a structured interview to evaluate the participants for the presence of PTSD and other possible comorbid psychiatric diagnoses and was chosen because it takes two thirds less time to administer.

### Selection and description of participants

Participants were adolescents who were recruited from lower-socioeconomic-status community schools around Cape Town. Inclusion criteria were age 13 to 18 years and a primary diagnosis from the MINI-KID of chronic or subthreshold PTSD secondary to a trauma that occurred at least 3 months prior to assessment for the study. Subthreshold PTSD was defined as: one or more re-experiencing symptoms, two or more avoidance symptoms, two or more arousal symptoms, and a total score of 11 or greater on the CPSS-I. This cut-off was chosen so as to capture those adolescents who do not meet the full criteria for PTSD but who are experiencing symptoms above threshold in the subclinical range.

Adolescents with conduct disorder were excluded. Participants seeking or requiring alternative treatments (e.g. medication) were also excluded. All other adolescents were eligible for participation. For example, this included adolescents with comorbid mood disorders, anxiety disorders, substance use disorders, and attention-deficit hyperactivity disorder (ADHD) provided that PTSD was deemed to be the primary disorder requiring treatment.

**Fig. 1:**
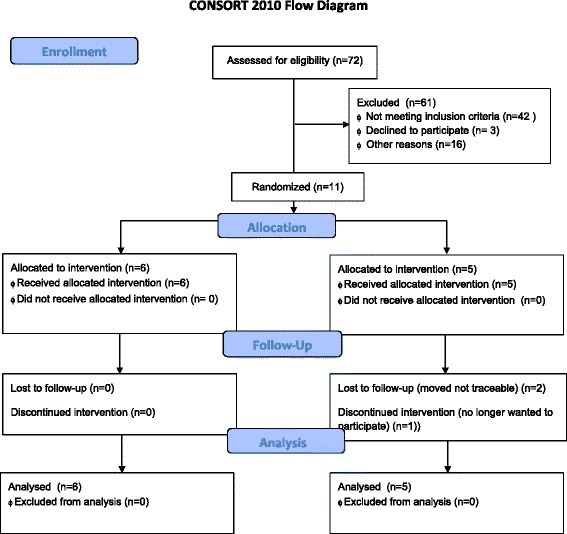
Consolidated Standards of Reporting Trials (CONSORT) 2010 flow diagram

Figure 1 reflects participant flow through the study and shows the recruitment rate and reasons for exclusion. Of all the initial potential participants who volunteered for this pilot, the majority completing the screening instrument did not report experiencing or witnessing a traumatic event or did not meet the minimum screening criteria set (CPSS-SR > 11). Of the 61 adolescents excluded, 42 did not meet the cut-off score criterion. Thirty-five did not report experiencing a trauma and seven experienced grief after loss of a significant caregiver and did not meet PTSD criteria. Three declined treatment when contacted for preassessment. Of the 16 participants listed under other reasons, 5 did not complete informed consent (parent or guardian), 2 adolescents were beyond the age limit for the study (below 18 years of age), and 9 adolescents’ parents were not contactable (despite numerous attempts to schedule a meeting).

### Procedure

The study was approved by the Health Research Ethics Committee of Stellenbosch University. Permission was also obtained from the Western Cape Education Department (WCED) to offer treatment at the participants’ schools. Permission from the WCED was conditional on not disclosing the identities of the participating schools. Participants for this study were recruited from three schools. All three schools’ principals agreed to allow the students at their schools to participate. Schools had approximately 900 students from grades 8 through 12. All schools were free-tuition schools and were located in low-socioeconomic-status areas. A recruiter addressed the children during assembly and in individual classes. The recruiter explained the definition of a traumatic event, described typical events that would be considered a traumatic experience, explained the diagnosis of PTSD and explained that this study aimed to compare the effectiveness of two treatments when administered by nurses within the community (school). Adolescents who thought that they could benefit from treatment completed a PTSD screening instrument in private. Prospective participants who indicated that they had experienced a trauma and whose scores were beyond the cut-off for inclusion were scheduled for a pretreatment assessment. For those who met the criteria, a preassessment interview was scheduled with the adolescent and their parent or guardian. The pre- and post-assessment interviews were conducted by two independent evaluators blind to treatment condition; one was an experienced psychiatric research nurse and the other a clinical psychologist experienced in research diagnostic interviewing.

All participants and their primary guardians gave written informed consent/assent, after reading the Consent/assent Forms that gave a full explanation on the nature of the study and what would be expected of participants. This was done in the presence of, and with the help of, one of the investigators. Questions and concerns were addressed before signing the documents. Participants then completed a 2- to 3-h baseline evaluation, comprising a clinical interview and self-report measures to assess eligibility and capture baseline information. The follow-up assessments, were 1.5 h in duration. Several of the measures administered during these lengthy evaluations will not be reported in this paper as they are beyond the scope of this pilot and feasibility study. Participants who met the criteria were randomised to receive either PE-A or SC using a parallel design and a permuted block procedure with six randomisations per block (1:1 ratio), generated with a computer randomisation schedule by a statistician prior to the beginning of enrollment. The primary investigator consulted the randomisation table and informed the counsellor of the type of treatment that the participant would be receiving.

## Assessment

Participants completed the self-report measure of PTSD (CPSS-SR) and of depression (BDI) prior to each treatment session. Participants also were administered the interview-based measure of PTSD (CPSS-I) and completed the CPSS-SR and BDI at all independent evaluations, which occurred before treatment, at mid-treatment, immediately after completion of treatment, and at 3, 6, and 12 months post treatment. Other measures included at the baseline evaluation were a demographic questionnaire, a diagnostic interview, psychopathology measures, cognition and emotion measures, and parent or guardian psychopathology measures. Participants completed all the measures except for the diagnostic information at all follow-up assessments. The analysis of these measures was not included here as it was beyond the scope of this paper and will be addressed in the main study.

Treatment sessions were video- and audio-recorded and reviewed during supervision to monitor treatment protocol adherence and to assist the nurse counsellors in treatment management. Audio-recordings were listened to by the participants as homework assignments. SC sessions required the counsellors to note at every session whether participants referred to the trauma experience.

## Treatment

PE-A was chosen as a trauma-focussed intervention that fulfills the recommendations for choosing a treatment to disseminate in a task-shifting community environment [14]. SC provides an active control for PE-A which is a plausible alternative treatment with similar dose and time-response characteristics. Variants of SC are widely used in rape crisis centres and other community settings to treat sexually abused children [25]. Moreover, SC was used as a control condition in several studies that evaluated PE for adult PTSD and related psychopathology [26]. The direct comparison of PE-A and SC is of considerable public importance from the point of view of cost-effectiveness and feasibility. Specifically, if PE-A is indeed superior to SC within a South African context, it would provide a compelling rationale to retrain therapists in this alternative treatment.

Both treatments were delivered by six nurses who were completing a 1-year advanced psychiatry diploma at Stellenbosch University. The nurses attended a 3-day PE-A training workshop (by JR and EY, both of whom are certified PE therapists and supervisors) and a 1-day SC training (by JR). The SC training was followed by additional 16 h of practical training. They received group supervision from one of the authors (JR) every week throughout the trial, during which very close attention was given to upcoming session preparation and emphasis on adherence to each of the protocols.

The PE-A program [27] consists of up to 14 weekly 60- to 90-min sessions. Treatment comprises eight modules. Homework exercises provide the opportunity for practice outside of the session. Module 1 includes presentation of the treatment rationale. Module 2 includes information gathering, identifying an index trauma, and conducting a breathing-retraining exercise. Module 3 presents common reactions to trauma. Module 4 includes discussion of the rationale for in vivo exposure (confronting trauma reminders in real life), creation of the in vivo hierarchy, and assignment of in vivo homework. Module 5 includes presentation of the rationale for imaginal exposure (revisiting and recounting the traumatic memory), conducting imaginal exposure for 15 to 45 min, and processing this revisiting experience. This module is repeated for two to five sessions. In module 6, the imaginal exposure focusses on the worst moments of the trauma. Module 6 is repeated for four to seven sessions. Module 7 focusses on generalisation of skills learned in treatment and on relapse prevention. Module 8 comprises a “final project”, such as making a collage detailing the trauma and the gains made in treatment.

The SC treatment consists of up to 14 weekly 60- to 90-min sessions of client-centred therapy [28]. SC is based on the Traumagenic Dynamics Model [29] of symptom formation after child sexual abuse and the Rogerian psychotherapy model [30]. SC sessions focus on establishing a trusting, empowering, and validating therapeutic relationship. Participants are allowed to choose when, how, and whether or not to address their trauma. In session 1, participants are oriented to SC.

Counsellors provide active listening, empathy, and encouragement to talk about feelings and express beliefs in the participant’s ability to cope. In sessions 4 and 8, participants are asked how they feel about their trauma. With this exception, participants direct the sessions. Counsellors made note of the frequency of the discussion of the trauma.

## Statistics

Stata version 13.0 (StataCorp., College Station, TX, USA) was used for data analysis. Analysis was performed on an intention-to-treat basis. A linear mixed model (LMM) was employed to analyse the relationship between CPSS or BDI and treatment arm over time and included all randomised participants. This statistical model allows one to impute the values for missing data. Time was included in the model as categorical. The interaction between time and treatment arm was also investigated.

## Results

As can be seen in Fig. 1, all of the participants in the PE-A group completed treatment (*n* = 6) and four of the five participants in the SC group completed treatment. The one participant in the SC treatment arm who terminated treatment early reported that her reason was that she felt better and did not require any further intervention. Two participants were lost to the 12-month follow-up due to relocation to an unknown address.

All participants were included in the analysis. Analysis was performed on an intention-to-treat basis. Data collection lasted from March 2013 to October 2014. The age of participants ranged from 14 to 18 years, with a mean age of 16 years. Ethnologically, there were six African (54.5%) and five Mixed Race (45.5%) participants. Participants were predominantly female (*n* = 10).

Objective indices of tolerability or acceptability were inadvertently omitted in this pilot study. One indication that both treatments seemed to be well-tolerated by the participants is based solely on the fact that there was only one dropout from the SC group and none from the PE-A group. Because formal fidelity measures were not available for the pilot we are limited with regard to reporting on deviations from either protocol fidelity except for the subjective observations made during the supervisory process. Accordingly, no deviations from either protocol were identified during the weekly supervision sessions. Review of the session notes of the SC group showed that minimal mention of the trauma or discussion of the trauma occurred. Two participants discussed the trauma that they had experienced once, two discussed the trauma twice and one did not mention the trauma at all. The exact time in minutes that each of the four participants spent revisiting trauma experiences was not provided in the treatment notes. However, in comparison to the group that received directed PE-A, which entailed a minimum of six sessions of imaginal exposure and between-session listening to it, as well as 7 weeks of in vivo exposures, the amount of focus on the trauma was minimal and incidental.

Table 1 summarises the PTSD and depression symptoms at baseline, post treatment assessment, and at 12-month follow-up and shows the between-treatment comparison. PTSD symptoms, as measured by the CPSS-I, significantly improved in the PE-A group from pre-treatment assessment to post-treatment assessment (*p* < 0.05), as well as at the 12-month follow-up (*p* < 0.05). The SC group also improved significantly from pre-treatment assessment to post-treatment assessment (*p* < 0.05) but did not maintain these gains at the 12-month follow-up. At the 12-month follow-up, CPSS-I scores were significantly lower in the PE-A group than in the SC group. The between-treatment difference in improvement from baseline to the 12-month follow-up was 16.9; 95% CI, 2.3–31.6; *p* = 0.023.

**Table 1:** Primary and secondary outcomes at baseline, post treatment, and at 12-month follow-up

	PE-A (*n* = 6)	SC (*n* = 5)	Between-treatment differences in improvement from baseline	*p* value
Primary outcome				
Interviewer-rated PTSD (CPSS-I), Mean (95% CI)				
Baseline	32.3 (26.5–38.2)	34.8 (28.4–41.2)		
Post treatment	2.8 (−3.0–8.7)	10.0 (1.8–18.1)	4.7 (−7.3–17.7)	0.4418
12-month	3.0 (−2.8–8.8)	22.4 (12.3–32.5)	16.9 (2.3–31.6)	0.0234
Secondary outcome				
Depression (BDI), mean (95% CI)				
Baseline	22.3 (14.5–30.1)	27.6 (19.1–36.1)		
Post treatment	2.1 (−5.6–10.0)	4.3 (−6.6–15.2)	−3.2 (−19.6–13.3)	0.7068
12-month	4.7 (−3.1–12.5)	32.7 (19.2–46.2)	22.7 (3.0–42.6)	0.0295

*BDI* Beck Depression Inventory, *CI* confidence interval, *CPSS-I* Child PTSD Symptom Scale–Interview, *PE-A* Prolonged Exposure Treatment for Adolescents, *PTSD* post-traumatic stress disorder, *SC* supportive counselling. Baseline and total sample data reflect the raw means. Means for the treatment conditions at the post treatment assessment and at 12-month follow-up and differences in improvement are taken from the linear mixed model growth curve analyses. The *p* value is for between-treatment differences in improvement

In both the PE-A and SC groups, depression (as assessed on the BDI) improved significantly at the post-treatment assessment compared to the pre-treatment assessment (*p* < 0.05). These gains were maintained at the 12-month follow-up (*p* < 0.05) for the PE-A group. At the 12-month follow-up, BDI depression scores were significantly lower in the PE-A group than in the SC group. The between-treatment difference in improvement from baseline to the 12-month follow-up was 22.7; 95% CI, 3.0–42.6; *p* = 0.030.

## Discussion

The outcome data of this small-sample-size pilot and feasibility study suggest that the PE-A protocol may be implemented in a South African context when administered by previously inexperienced counsellors within a school setting. It is encouraging that completely treatment-delivery-naïve nurses can be trained in a relatively short time to administer treatment under supervision. If these preliminary results are substantiated in the study proper that is currently underway, this would bode well for dissemination of treatment for PTSD in a third-world, resource-impoverished setting.

The current study’s preliminary results are not directly comparable with the studies of similar nature that were mentioned earlier [19, 20]. As a pilot, it consisted of a small sample size and was, therefore, not adequately powered. Despite that, the preliminary results of this pilot study were similar to those in the studies with larger sample sizes in that participants’ PTSD and depression scores improved significantly in the PE-A treatment arm and these gains were maintained at the 12-month follow-up. An interesting difference between the current pilot study and the studies cited above was that the PE-A group did not show superiority to the SC group at the post-treatment assessment, but did so at the 12-month follow-up. The results for the SC group suggest that this treatment may be helpful in relieving symptoms of PTSD and depression, at least initially. However, the lack of maintenance at 12-month follow-up would require further investigation in an adequately powered RCT.

All participants in our study were seen by the independent evaluator after five sessions, directly after completion of treatment and 3, 6, and 12 months after completion of treatment. This was done in an effort to maintain contact with participants which facilitated retention; these contacts, as well as enabling the notification of any change in address, provided motivation for the participants to return for the assessments. During the active treatment phase, counsellors were asked to schedule their appointment with the participants for the next week during the session. Participants and their parents or guardians also had the contact details of the primary investigator (JR). Most participants had mobile phones and could contact their counsellor in this way between sessions. Telephone messaging was primarily used for notification of a change in appointments. We believe that these measures have significantly contributed to the low dropout rate in this pilot study and we are implementing them in the study proper.

As mentioned earlier, the counsellors offering the treatment in this study were nurses who were taking a 1-year advanced psychiatry diploma course. As a consequence, they only had 1 day a week available to participate in the study and were not able to see more than three adolescent participants each per week. This led to a higher ratio of counsellors to participants. This issue is being addressed in the study proper, where new counsellors will be trained every year and none of the participating counsellors will treat more than four participants each. The fact that every year newly trained counsellors will be administering treatment to participants will allow us to evaluate whether truly inexperienced nurses can effectively offer treatment to adolescents with PTSD.

This feasibility and pilot study is unique in that participants were recruited and enrolled at their schools and then received treatment at their schools on a weekly basis. On the one hand, this strategy may have biased the sample in that it may have excluded adolescents, who, due to the effects of their PTSD, are unable to attend school. On the other hand, it created an opportunity and ease of participation for those who would otherwise have lacked the means or family support to attend treatment sessions once they had gone home from school. This brings treatment to the adolescent community in the school setting and is in line with a move towards task-shifting and making treatments available within the communities’ natural milieu [1].

Several limitations should be noted. One limitation of the study is the small sample size which naturally restricts the generalisability of its findings. It does, however, provide a point of departure for testing the effectiveness of PE-A delivered by community health nurses in the context of a larger-scale RCT in a resource-limited environment. Due to the loss of three participants in the SC group at the 12-month follow-up, the results should be interpreted with caution. Our main study is currently in its final year and active treatment will be completed by the end of 2016. Another limitation of the pilot study is the gender imbalance, where most of the participants were female, again limiting the generalisability across genders. This may be due to the fact that female adolescents seem to be more likely to volunteer for treatment or that they are more likely to present with PTSD. One other limitation of this study is that we did not include treatment satisfaction measures which would provide a clearer assessment of the palatability of the treatments. This limits the conclusion about whether the protocols were deemed acceptable or not by the participants. This omission is being remedied in the main study.

An interesting phenomenon occurred during this pilot study, creating relatively high exclusion rates. This was due to the fact that the large number of youngsters who volunteered to participate in the study seemed to be related to the difficulty that adolescents in this community experience in accessing mental health care generally. Several adolescents appeared to volunteer their participation in the study due to this access difficulty. Consequently, we have initiated a qualitative investigation of adolescents in the main study to capture both their experiences of accessing mental health care in their community as well as of participating in this treatment study. In undertaking recruitment for the study proper, the recruiters have modified their presentation at the schools further emphasising that the study is a treatment study only for PTSD and that that is a consequence of exposure to a traumatic event. The coordinator at the school was also briefed in more detail to prevent inappropriate referrals to the study. We have since seen a reduction in referrals that are not meeting the inclusion criteria for the study proper.

## Conclusion

The small-sample feasibility pilot study demonstrated that the PE-A and SC treatment models can be delivered by nurses to adolescents within a school setting in a South African context. With the caveat of the noted study limitations, both PE-A and SC resulted in reduced independent evaluator interviewer-assessed PTSD severity scores and self-reported depression scores when measured immediately after completion of treatment, and there was maintenance of those gains in the PE-A group at the 12-month follow-up assessment. Based on the experience during the feasibility pilot study, several lessons were learned and changes made accordingly for the main trial. These included improving the referral process to reduce the number of inappropriate referrals (such as persons who did not meet the criteria for PTSD) and to assess for treatment satisfaction.

## Trial status

This article was first submitted on 7 October 2015. The feasibility study started recruitment in January 2013. Seventy-two potential participants indicated an interest in participating in the study. Eleven participants met the eligibility criteria and were randomised to treatment. Treatment was completed during 2013. One-year follow-up was completed towards the end of 2014.

## Abbreviations

BDI: Beck Depression Inventory
CPSS-I: Child Post-traumatic stress disorder Symptom Scale–Interview
K-SADS-PL: Schedule for Affective Disorders and Schizophrenia for School-Aged Children–Present and Lifetime Version
MINI-KID: The Mini International Neuropsychiatric Interview for Children and Adolescents
PE-A: Prolonged Exposure Treatment for Adolescents
PTSD: Post-traumatic stress disorder
RCT: Randomised control trial
SC: Supportive counselling
